# Firearm injury prevention counseling for patients with traumatic brain injury: a survey of brain injury physicians

**DOI:** 10.3389/fneur.2023.1237095

**Published:** 2023-08-24

**Authors:** Ian Ramsay, Natalia Del Mar Miranda-Cantellops, Oliver Acosta, Lauren T. Shapiro

**Affiliations:** ^1^MD/MPH Program, University of Miami Leonard M. Miller School of Medicine, Miami, FL, United States; ^2^Brain Injury Fellowship Program, Shepherd Center, Atlanta, GA, United States; ^3^Physical Medicine & Rehabilitation Residency Program, University of Miami/Jackson Health System, Miami, FL, United States; ^4^Department of Physical Medicine & Rehabilitation, University of Miami Leonard M. Miller School of Medicine, Miami, FL, United States

**Keywords:** traumatic brain injury, firearms, gun violence, patient safety, brain injury medicine

## Abstract

**Background:**

Survivors of traumatic brain injury are at increased risk for firearm-related injuries, including suicide.

**Aims:**

To determine current practices of Brain Injury Medicine (BIM) physicians and their rehabilitation teams in assessing patients’ access to firearms and in providing firearm safety education, and the impact of having received training on this topic on physicians’ likelihood of inquiring about patients’ access to firearms.

**Methods:**

14-item web-based cross-sectional survey of 86 U.S. physiatrists board-certified in BIM.

**Results:**

81% of respondents indicated they believe BIM physicians should counsel their patients on firearm safety but only 12.9% reported always doing so. Fifteen percent reported always inquiring about their patients’ access to firearms. 88.2% indicated having never received formal training on firearm injury prevention counseling. Physicians who received such training had 7.5 times higher odds of reporting at least sometimes inquiring about patients’ access to firearms than those who were not trained [95% confidence interval (1.94, 28.64)]. They also had 5.7 times higher odds for reporting being at least moderately comfortable providing patients firearm safety counseling [95% CI: (1.39, 23.22)].

**Conclusion:**

While most BIM specialists who responded to this survey believe they should counsel patients on firearm safety, few always or usually do so. Moreover, most do not routinely inquire about their patients’ access to firearms. The provision of firearm injury prevention training to BIM physicians was strongly associated with an increased likelihood they will inquire about their patients’ access to guns and with an improved comfort level in providing counseling on this subject matter.

## Introduction

In the United States, firearm-related injuries present a critical public health issue and are a significant contributor to the incidence of traumatic brain injury (TBI) and TBI-related deaths (1, 2). They accounted for 2.2% of adults with TBI presenting to U.S. trauma centers between 2003–2012, a time during which 54.6% of these gunshot injuries were fatal (1). The risk for firearm-related injury and death is increased when firearms are present in one’s home. The majority of accidental shooting deaths occur in a home environment, and the presence of a gun increases the potential for altercations to become fatal. Having a gun in the home is also an important risk factor for suicide, even among members of the household without prior history of mental illness or substance abuse (3). As 44 % of U.S. adults live in a home with one or more guns (4), firearms should be viewed as a common household hazard.

Multiple physician organizations have called for measures aimed at the prevention of firearm-related injuries (5). In its position paper on reducing firearm injuries and deaths, the American College of Physicians encouraged doctors to have discussions with their patients regarding the risks associated with having a firearm in their household and the means by which they may reduce these risks (6). Nevertheless, the results of the National Firearms Survey revealed that fewer than 10% of adults residing in a home with a firearm reported having ever discussed the topic with a clinician (7).

Little is known about the practice of Brain Injury Medicine (BIM) physicians regarding their assessment of patients’ access to guns and their provision of firearm safety counseling, though they provide care to a patient population at increased risk for both suicide and unintentional death by firearm (8). There are a multitude of factors that likely contribute to this elevated risk among persons with TBI. First, impairments resulting from their injuries may impact their ability to safely use a firearm. These include impairments in mobility, vision, and balance, as well as judgment, executive function, and emotional regulation (9). Another important factor concerns how persons with TBI store their weapons. Safe storage is associated with a lower risk for firearm-related morbidity and mortality (10). Among military service members who own firearms, those with suspected TBI are significantly less likely to practice safe storage than those without suspected TBI (11). A key consideration for those who sustained their injuries from a firearm assault is their risk for revictimization. In a retrospective cohort study of persons treated for nonfatal firearm assault injuries (not limited to TBI) in California, more than 3% sustained one or more additional nonfatal firearm assault injuries and 1% died as a result of a subsequent homicide with a firearm (12).

Far more persons with TBI, however, die by suicide with a firearm. Individuals with a history of TBI are at significantly higher risk for death by suicide than those without such history. Among persons with TBI, risk factors for suicide include suicidal ideation, as well as comorbid depression, substance use disorders, anxiety disorder, and post-traumatic stress disorder. Insomnia may also be an important risk factor (13). Firearms are the most common means of death by suicide among persons with TBI (14). Among firearm suicide cases, the odds of having had a TBI is 23.53 times higher than it is among controls who did not die by suicide (15). It is important to note that nearly 1 in 5 suicides among persons with TBI occur among those with a history of military service (14), and that individuals with a history of such service have the highest rates of firearm suicide (16).

This study serves as a preliminary exploration of the perceptions of the need for and practices providing firearm injury prevention counseling to persons with TBI among BIM physicians. It also aims to determine the associations between having received training in firearm injury prevention on the likelihood of BIM physicians inquiring about patients’ access to firearms and on their comfort level providing counseling on this topic.

## Methods

This study was submitted for review to the University of Miami Institutional Review Board (IRB ID# 20210668), which determined it met criteria for an exemption. Survey participants were advised that completion of the survey indicated agreement to participate in this study.

### Survey development and content

The study team reviewed prior studies evaluating physicians’ perceptions of the need for and provision of firearm injury prevention counseling, including the 2020 Council of Academic Family Medicine’s Educational Research Alliance survey of family physicians (17), which helped inform this survey’s development. No prior published studies were conducted among BIM physicians, and accordingly, this survey’s items were created anew.

The survey consisted of 14 questions. Respondents were asked to identify their primary specialty, whether they primarily see adult or pediatric patients, the type of facilities in which they provide care, and the state in which their practice or institution is located. They were asked about their beliefs as to whether BIM physicians should counsel their patients with TBI on firearm safety, their comfort level doing so, their training in providing such education, and their team’s current practices regarding inquiry about their patients’ access to firearms and in providing firearm injury prevention counseling. Lastly, they were asked to provide any additional topical comments they wished to share.

### Study participants and invitation methods

Electronic invitations to participate were sent to physicians board-certified in the subspecialty of Brain Injury Medicine identified via a search of the American Board of Physical Medicine and Rehabilitation (ABPMR)‘s website (18). Physicians located outside of the U.S. and its territories were excluded. At the time the survey was conducted, 667 physicians nationwide met the inclusion criteria. The physicians for whom the study team was able to locate an email address received an invitation to participate, with the survey link, via email, using REDCap distribution tools. A reminder email was sent eight days following the initial invitation to those who had not yet responded.

Of the BIM physicians for whom an email address was not located, the majority had messaging enabled on the Doximity messaging platform, through which the study team sent them invitations to participate along with the survey link. Doximity is an online community which enables secure electronic messaging between health care professionals (19). These individuals did not receive a reminder invite, as whether they responded could not be tracked.

Participants did not receive an incentive to respond. The survey remained open for a two-week period in August 2021.

### Data collection

Survey data were collected and managed using REDCap electronic data capture tools hosted at the University of Miami. REDCap (Research Electronic Data Capture) is a secure web-based software platform designed to support data capture for research studies, providing (1) an intuitive interface for validated data capture; (2) audit trails for tracking data manipulation and export procedures; (3) automated export procedures for seamless data downloads to standard statistical packages, and (4) procedures for data integration and interoperability (20, 21).

### Missing data

Participants had the option of not responding to items on the survey. All but two items received at least 84 responses. Participants who did not respond to a question were excluded from the sample size for that item.

### Statistical methods

The frequency of inquiring about patients’ access to firearms was coded as one of three options: always/usually, sometimes, and rarely/never. The participants’ level of comfort in providing firearm safety counseling was categorized as high (7 – 10), moderate (4 – 6), or low (0–3). Fisher’s exact test and ordinal logistical regression were used to examine the association of receiving training in firearm injury prevention with the reported frequency of inquiring about patients’ access to firearms and comfort levels providing firearm safety counseling. The proportional odds assumption was checked and not violated. All analyses were conducted using SAS version 9.4.

## Results

Responses were received from 86 U.S. BIM physicians, all physiatrists, practicing in 30 states and Puerto Rico. Table 1 summarizes participant characteristics, and Table 2 displays the distribution of responses on items pertaining to the frequency with which the physicians’ teams inquire about their patients’ access to guns and with which they provide counseling on firearm injury prevention. Of note, 81% of respondents agreed that BIM physicians should counsel their patients on firearm injury prevention, but only 12.9% reported always doing so, 95% CI [6.6, 22%]. 15% reported always inquiring about access to firearms among their patients with TBI, 95% CI [8.3, 24.4%].

**Table 1:** Characteristics of responding physicians.

Characteristic	# of Respondents Selecting Option
Primary specialty – Physical Medicine & Rehabilitation (*n* = 86)	86 (100%)
Primary patient population by age group (*n* = 86)	
Adults	72 (83.7%)
Children	12 (14.0%)
Approximately equal numbers of children and adults	2 (2.3%)
Type of facility/facilities in which they provide care (*n* = 86)	
Academic medical center	56 (65.1%)
Community hospital	26 (30.2%)
Multispecialty group	12 (14.0%)
Veterans Affairs Medical Center	8 (9.3%)
Private office	7 (8.1%)
Military healthcare facility	0 (0%)
Location of practice/institution – top 5 responses (*n* = 86)	
Texas	13 (15.1%)
California	9 (10.5%)
Florida	6 (7.0%)
New York	6 (7.0%)
Ohio	6 (7.0%)

**Table 2:** Fisher’s test contigency tables for associations with training in firearm injury prevention.

		Formal training in firearm injury prevention		
	*n*	Yes (*n* = 10)	No (*n* = 75)	*p*-value
Frequency of inquiring about patients’ access to firearms	86			
Always/Usually	20	6 (60.0%)	13 (17.3%)	0.007
Sometimes	28	3 (30.0%)	25 (33.3%)	1.0000
Rarely/Never	38	1 (10.0%)	37 (49.4%)	0.021
Comfort (on scale from 1 to 10) providing firearm safety counseling^*^	84			
High (7–10)	27	7 (70.0)	20 (27.4)	0.012
Moderate (4–6)	36	2 (20.0)	34 (46.6)	0.175
Low (0–3)	21	1 (10.0)	19 (26.0)	0.438

^*^Participants were asked, “On a scale of 0–10, how comfortable are you in counseling patients on firearm safety? (0 = not at all comfortable, 10 = very comfortable).”

**Figure 1:**
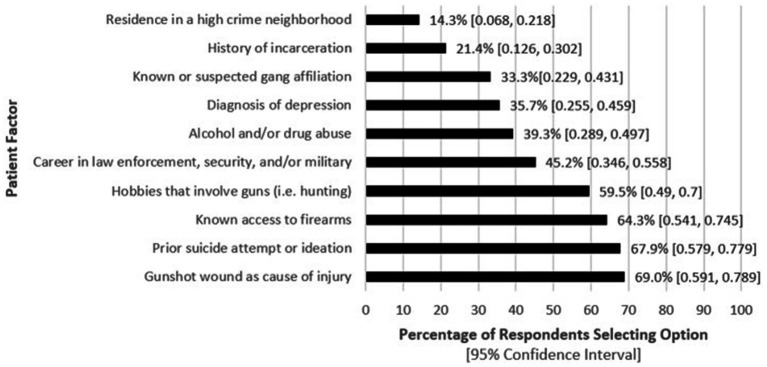
Patient factors reported by physician respondents that increase the likelihood they will counsel TBI patients on firearm safety (*n* = 84).

Respondents reported several patient factors increase their likelihood of inquiring about their access to firearms, with the most common being gunshot wound as the cause of injury (69%) and prior suicide attempts or ideation (67.9%), with 95% CIs of [58, 78.7%] and [56.8, 77.6%], respectively. These results are shown in Figure 1.

**Figure 2:**
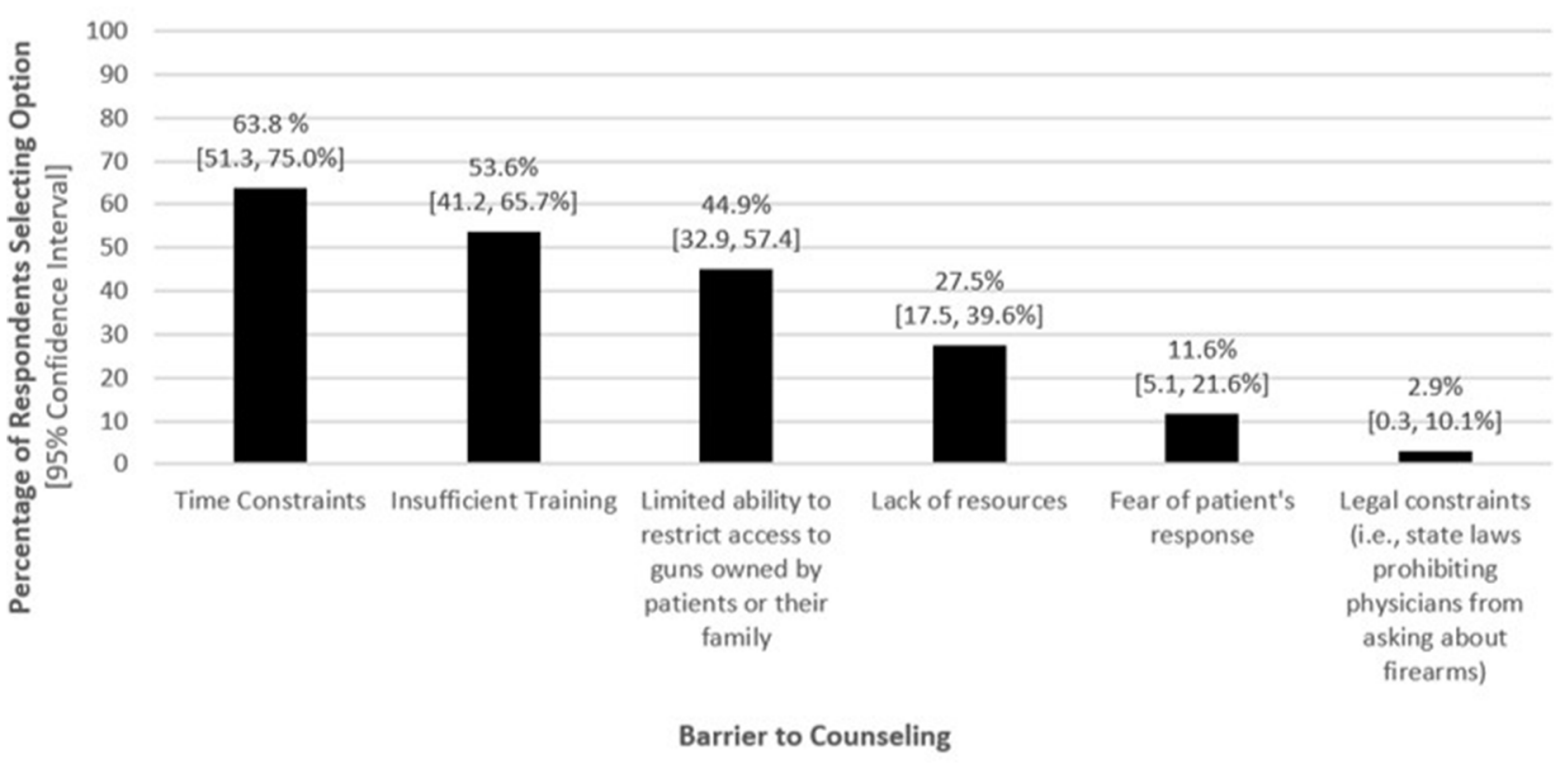
Self-reported factors preventing physicians from counseling TBI patients on firearm safety (*n* = 69).

Figure 2 illustrates factors that reportedly prevent the respondents and/or their team from providing this counseling, with time constraints and insufficient training each selected by more than half of the respondents as barriers.

88.2% of survey participants indicated having never received formal training on firearm injury prevention or counseling, 95% CI [79.4, 94.2%]. Physicians who received training in firearm injury prevention had 7.5 times higher odds of reporting at least sometimes inquiring about patients’ access to firearms than those who did not receive training, 95% CI [1.94, 28.64]. They also had 5.7 times higher odds for reporting at least moderate comfort levels (4 or higher on a 0–10 scale) providing firearm safety counseling than those who did not, 95% CI [1.39, 23.22].

70% of respondents reported having asked a friend or family member of a patient with TBI to remove a firearm from the home, 95% CI [59.7, 80%]. 14% of respondents indicated having had a patient with TBI die by suicide by firearm, 95% CI [7.4, 23.11%].

Twenty-eight respondents provided additional comments. Three comments underscored the need for more training on this topic, while two addressed why they felt the topic often gets neglected in clinical settings. Seven comments pertained to the respondents’ personal criteria for deciding to whom such education should be provided.

## Discussion

Most surveyed believe BIM specialists should counsel patients on firearm safety, but the majority do not often or always do so. Additionally, most do not routinely inquire about the presence of firearms in their patients’ homes. Respondents reported being more likely to counsel patients with conditions known to increase the risk for suicide among persons with TBI (e.g., depression, substance use disorders, and/or a prior suicide attempt or ideation (13)). Many BIM physicians also reported being more likely to provide such counseling to those whose careers and/or hobbies involve firearms. Importantly, many respondents indicated being more likely to provide such counseling to patients who had sustained firearm-related TBIs. This group may be particularly high-risk for subsequent firearm-related injuries. Survivors of firearm injuries are at significantly elevated risk of carrying firearms and of higher levels of post-traumatic stress disorder symptoms when compared to survivors of other mechanisms of traumatic injuries (22). Moreover, survivors of firearm assaults are at increased risk for recurrent assaultive firearm injury and death (12).

Most BIM physicians surveyed reported having asked a friend or family member of a patient with a TBI to remove firearms from the home. It is noted that some states have legal barriers to the temporary transfer of a firearm to a friend or family member, including the potential need for a background check or completion of safety training certification by the individual to whom it is being transferred (23).

Only two respondents, both practicing in Florida, indicated that state laws prevent them from counseling patients about firearm safety. Florida passed the Firearm Owners’ Privacy Act in 2011, which prohibited physicians from routinely inquiring about their patients’ ownership of firearms, but the U.S. Court of Appeals overturned it in 2017 (24). A subsequent survey of faculty physicians at the University of Florida revealed that many were not aware that the restrictions on physicians discussing firearms with their patients had been ruled unconstitutional and were no longer in effect (25).

Resources, such as pamphlets or web applications, that educate patients and caregivers about the impact of sequelae of TBI on one’s ability to safely store, load, and utilize a firearm, may allow health care teams to provide recommendations to mitigate one’s risk of firearm-related injury without the time burden that providing one-on-one counseling entails. Moreover, such materials are likely to receive a neutral or positive response from patients and their family members (26–28). It is noted that educational resources on this topic have been made available by the Department of Veterans Affairs, including an informative website and a toolkit addressing safe firearm storage (29, 30).

More extensive counseling on firearm safety may be necessary for patients at highest risk for firearm-related suicide or injuries, and it is vital to address physicians’ lack of training and comfort in providing this education. A prior study found that family physicians who received formal training on firearm safety counseling reported greater comfort in asking their patients about firearms (17). A survey of North Carolina physicians revealed that few had attended continuing medical education events on gun violence, but participation in such events was strongly associated with providing patient firearm counseling often or very often (31). Accordingly, the development of educational programs for health care professionals caring for persons with TBI addressing firearm injury prevention may be an appropriate strategy to improve their training and comfort in the provision of this education and the likelihood they will provide it.

## Limitations

Among this study’s limitations is the lack of responses from physicians practicing in the states with the highest estimated average household firearm ownership rates (Montana, Wyoming, Alaska, Idaho, and West Virginia) (32). This is likely secondary to a dearth of physicians meeting inclusion criteria in those states, as there were only 3 physicians board-certified in BIM in those states combined at the time this study was conducted (18). Nevertheless, this may impact the generalizability of the findings.

Potentially important physician characteristics, such as age, gender, and gun ownership, were not inquired about. There is evidence that physicians who own firearms may be more likely to counsel patients about firearm safety (33).

Lastly, BIM physicians often provide care within multidisciplinary rehabilitation teams, and some may be unaware of the screening and counseling provided by other team members.

## Conclusion

Individuals with TBI have a significantly higher risk of firearm-related death. Although most surveyed BIM physicians believe they should offer firearm injury prevention counseling to their patients, they perceive barriers to doing so. Only a small minority (less than 12%) have received firearm safety training, but those who have are more likely to inquire about firearm access and feel comfortable educating patients on this matter. Creating firearm injury prevention training programs for rehabilitation professionals caring for persons with TBI, along with educational resources for patients and caregivers, could enhance awareness and prevent firearm-related injuries in this vulnerable group.

## Data availability statement

The raw data supporting the conclusions of this article will be made available by the authors, without undue reservation.

## Ethics statement

The studies involving humans were approved by University of Miami Miller School of Medicine. The studies were conducted in accordance with the local legislation and institutional requirements. The ethics committee/institutional review board waived the requirement of written informed consent for participation from the participants or the participants’ legal guardians/next of kin because exempt (low risk, survey of physicians).

## Author contributions

IR, NM-C, and LS contributed to the conception and design of the survey and study. IR, NM-C, OA, and LS wrote sections of the manuscript. All authors contributed to the article and approved the submitted version.

## Conflict of interest

LS holds stock in Doximity. This platform was used to make contact with brain injury specialists for whom we were unable to locate an email address, though ultimately, we received only one survey response via this method.

The remaining authors declare that the research was conducted in the absence of any commercial or financial relationships that could be construed as a potential conflict of interest.

## Publisher’s note

All claims expressed in this article are solely those of the authors and do not necessarily represent those of their affiliated organizations, or those of the publisher, the editors and the reviewers. Any product that may be evaluated in this article, or claim that may be made by its manufacturer, is not guaranteed or endorsed by the publisher.
