# Supplementary material for: Firearm injury prevention counseling for patients with traumatic brain injury: a survey of brain injury physicians
Source: Front Neurol. 2023 Aug 24;14:1237095. doi: 10.3389/fneur.2023.1237095 (PMC10484094; doi:10.3389/fneur.2023.1237095)
Supplement: Supplementary file 1 [file Data_Sheet_1.PDF]

Survey on Assessing Access to Firearms and the Provision of Firearm Injury Prevention Education for Patients with Traumatic Brain Injury

We are studying the current practices of brain injury subspecialists with regards to assessing access to firearms among their patients with traumatic brain injury (TBI) as well as providing them counseling on firearm injury prevention. You are being asked to participate in this research study because you have been identified as a brain injury subspecialist.

If you agree to be in our study, we will ask you to agree to do 1 brief online survey (below). Completion of the survey indicates agreement to participate in this study. You do not have to be in the study if you do not want to; it is your choice and completely voluntary. You can change your mind at any time and there will be no penalty. Participants will not receive any payment or compensation for completion of this survey.

You do not have to share any information that you are not comfortable sharing. You can stop participating in the survey at any time. Your responses will remain confidential and anonymous. There are no known risks to participating in this study. You will not benefit directly from being in this research study. If you agree to participate in this survey study, please answer the questionnaire as best as you can. It should take no more than 10 minutes to complete the survey. Please only answer this survey one time.

If you have any questions about this study, please contact Dr. Lauren Shapiro at lxs973@med.miami.edu or (305)243-6605. If you have any complaints, suggestions, or questions about your rights as a research volunteer, please contact the Human Subject Research Office at the University of Miami at 305-243-3195 or send an email to hsro@miami.edu.

Please complete the survey below.

Thank you!

---

1) What is your primary specialty?

- ◯ Physical Medicine and Rehabilitation
- ◯ Emergency Medicine
- ◯ Family Medicine
- ◯ Internal Medicine
- ◯ Neurology
- ◯ Pediatrics
- ◯ Psychiatry

---

2) Do you primarily see adult or pediatric patients?

- ◯ Adults
- ◯ Children
- ◯ Approximately equal numbers of children and adults

---

3) In what type of facility or facilities do you provide clinical care? (Select all that apply)

- ☐ Community hospital
- ☐ Academic medical center
- ☐ Military healthcare facility
- ☐ Veterans Affairs Medical Center
- ☐ Private office
- ☐ Multispecialty group

---

4) In what state is your practice/institution located? (Select from drop-down)

- ◯ Alabama
- ◯ Alaska
- ◯ Arizona
- ◯ Arkansas
- ◯ California
- ◯ Colorado
- ◯ Connecticut
- ◯ Delaware
- ◯ District of Columbia
- ◯ Florida
- ◯ Georgia
- ◯ Hawaii
- ◯ Idaho
- ◯ Illinois
- ◯ Indiana
- ◯ Iowa
- ◯ Kansas
- ◯ Kentucky
- ◯ Louisiana
- ◯ Maine
- ◯ Maryland
- ◯ Massachusetts
- ◯ Michigan
- ◯ Minnesota
- ◯ Mississippi
- ◯ Missouri
- ◯ Montana
- ◯ Nebraska
- ◯ Nevada
- ◯ New Hampshire
- ◯ New Jersey
- ◯ New Mexico
- ◯ New York
- ◯ North Carolina
- ◯ North Dakota
- ◯ Ohio
- ◯ Oklahoma
- ◯ Oregon
- ◯ Pennsylvania
- ◯ Puerto Rico
- ◯ Rhode Island
- ◯ South Carolina
- ◯ South Dakota
- ◯ Tennessee
- ◯ Texas
- ◯ Utah
- ◯ Vermont
- ◯ Virginia
- ◯ Washington
- ◯ West Virginia
- ◯ Wisconsin
- ◯ Wyoming

---

5) How often do you or a member of your treatment team inquire about access to firearms among your patients with traumatic brain injury (TBI)?

- ◯ Always
- ◯ Usually
- ◯ Sometimes
- ◯ Rarely
- ◯ Never

6) What patient factors, if any, increase the likelihood you or a member of your treatment team would inquire about your patients' access to firearms? (Select all that apply)

- ☐ Alcohol and/or drug abuse
- ☐ Career in law enforcement, security, and/or military
- ☐ Diagnosis of depression
- ☐ Gunshot wound as cause of injury
- ☐ History of incarceration
- ☐ Hobbies that involve guns (i.e. hunting)
- ☐ Known access to firearms
- ☐ Known or suspected gang affiliation
- ☐ Prior suicide attempt or ideation
- ☐ Residence in a high crime neighborhood

7) How often do you or a member of your team counsel patients with TBI on firearm safety?

- ◯ Always
- ◯ Usually
- ◯ Sometimes
- ◯ Rarely
- ◯ Never

8) What, if any, factors prevent you or your team from counseling patients with TBI on firearm safety? (Select all that apply)

- ☐ Fear of patients' response
- ☐ Insufficient training
- ☐ Lack of resources
- ☐ Legal constraints (i.e. state laws prohibiting physicians from asking about firearms)
- ☐ Limited ability to restrict access to guns owned by patients or their family
- ☐ Time constraints

9) Have you ever received formal training on firearm injury prevention/counseling?

- ◯ Yes
- ◯ No

10) On a scale of 0-10, how comfortable are you in counseling patients on firearm safety? (0= not at all comfortable, 10= very comfortable)

Not at all comfortable — Very comfortable

(Place a mark on the scale above)

11) Have you ever asked a friend or family member of a patient with TBI to remove firearms from the home?

◯ Yes ◯ No

12) Have you ever had a patient with history of TBI die by suicide by firearm?

◯ Yes ◯ No ◯ Not sure

13) Do you believe brain injury specialists should counsel their patients on firearm safety?

○ Yes ○ No ○ Not sure

14) Do you have any additional comments that you would like to share on the topic of firearm injury prevention and counseling for patients with TBI?

____
